# Concentrations of the Sunscreen Agent Benzophenone-3 in Residents of the United States: National Health and Nutrition Examination Survey 2003–2004

**DOI:** 10.1289/ehp.11269

**Published:** 2008-03-21

**Authors:** Antonia M. Calafat, Lee-Yang Wong, Xiaoyun Ye, John A. Reidy, Larry L. Needham

**Affiliations:** Division of Laboratory Sciences, National Center for Environmental Health, Centers for Disease Control and Prevention, Atlanta, Georgia, USA

**Keywords:** benzophenone-3, biomonitoring, exposure, human, NHANES 2003–2004, sunscreen, urine

## Abstract

**Background:**

The capability of benzophenone-3 (BP-3) to absorb and dissipate ultraviolet radiation facilitates its use as a sunscreen agent. BP-3 has other uses in many consumer products (e.g., as fragrance and flavor enhancer, photoinitiator, ultraviolet curing agent, polymerization inhibitor).

**Objectives:**

Our goal was to assess exposure to BP-3 in a representative sample of the U.S. general population ≥ 6 years of age.

**Methods:**

Using automated solid-phase extraction coupled to high-performance liquid chromatography–tandem mass spectrometry, we analyzed 2,517 urine samples collected as part of the 2003–2004 National Health and Nutrition Examination Survey.

**Results:**

We detected BP-3 in 96.8% of the samples. The geometric mean and 95th percentile concentrations were 22.9 μg/L (22.2 μg/g creatinine) and 1,040 μg/L (1,070 μg/g creatinine), respectively. Least-square geometric mean (LSGM) concentrations were significantly higher (*p* ≤ 0.04) for females than for males, regardless of age. LSGM concentrations were significantly higher for non-Hispanic whites than for non-Hispanic blacks (*p* ≤ 0.01), regardless of age. Females were more likely than males [adjusted odds ratio (OR) = 3.5; 95% confidence interval (95% CI), 1.9–6.5], and non-Hispanic whites were more likely than non-Hispanic blacks (adjusted OR = 6.8; 95% CI, 2.9–16.2) to have concentrations above the 95th percentile.

**Conclusions:**

Exposure to BP-3 was prevalent in the general U.S. population during 2003–2004. Differences by sex and race/ethnicity probably reflect differences in use of personal care products containing BP-3.

Benzophenone-3 [2-hydroxy-4-methoxy-benzophenone, oxybenzophenone (BP-3)], a commonly used sunscreen agent that absorbs and dissipates ultraviolet radiation, is used in a variety of cosmetic products (Gonzalez et al. 2006; National Library of Medicine 2007; Rastogi 2002). BP-3 also has been used as ultraviolet stabilizer in plastic surface coatings for food packaging to prevent polymer or food photodegradation (Suzuki et al. 2005) and is approved by the U.S. Food and Drug Administration as an indirect food additive.

Human exposure to BP-3 has not been associated with adverse health effects, and acute toxicity from BP-3 is low. However, results from animal studies—primarily dietary studies that affected body weight gain—showed alterations in liver, kidney, and reproductive organs in rats and mice administered BP-3 dermally and orally (National Toxicology Program 1992). Although the maximum dose that could be administered dermally was similar to the lowest orally administered dose, which produced little systemic toxicity, these results suggested that oral and dermal exposure routes might affect the animals similarly (National Toxicology Program 1992). BP-3 also shows estrogen-like activity *in vitro* and *in vivo* (Schlumpf et al. 2001, 2003, 2004a, 2004b; Suzuki et al. 2005), although in one study BP-3’s estrogenic activity was observed only in the presence of a rat liver preparation, suggesting metabolic activation of BP-3 (Morohoshi et al. 2005). BP-3 can also display antiandrogenic activity *in vitro* (Ma et al. 2003; Schreurs et al. 2005). Thus, BP-3 might exhibit endocrine-disrupting action via both mechanisms in animals. Therefore, *in vivo* effects due to these combined activities should be further investigated.

The focus of pharmaceuticals and ingredients in personal care products, including organic sunscreen agents, as environmental pollutants is increasing because these compounds may enter the aquatic environment not primarily as a result of manufacturing practices but from their steady and widespread use in human and veterinary daily activities. Furthermore, little is known about the potential hazards associated with recurring human or ecologic exposures to these synthetic substances, many of which are bioactive (Daughton 2002; Daughton and Ternes 1999). BP-3, one of these substances, has been detected in surface waters (Balmer et al. 2005; Cuderman and Heath 2007), drinking water (Loraine and Pettigrove 2006; Stackelberg et al. 2004), and wastewater [Balmer et al. 2005; Centers for Disease Control and Prevention (CDC) 2003; Loraine and Pettigrove 2006] in North America and in Europe.

The widespread inclusion of sunscreen agents in personal care and consumer products (Gonzalez et al. 2006; National Library of Medicine 2007; Rastogi 2002) increases the potential for human exposure to BP-3. Data support the absorption of BP-3 through human skin (Gonzalez et al. 2006; Hayden et al. 2005; Janjua et al. 2004; Jiang et al. 1999; Sarveiya et al. 2004). Application of some of these products to large areas of the body and frequent reapplication increase the daily systemic absorption of BP-3. In some cases, as much as 10% of the applied dose can be absorbed (Jiang et al. 1999).

Like many xenobiotics, BP-3 undergoes phase I and phase II biotransformations. In rats, after oral and dermal administrations of 100 mg BP-3/kg body weight (Kadry et al. 1995; Okereke et al. 1993, 1994, 1995), the parent compound and three oxidative metabolites (2,4-dihydroxylbenzophenone, 2,2′-dihydroxy-4-methoxybenzophenone, and 2,3,4-trihydroxybenzophenone) were detected in plasma, tissues, and urine. Urine was the major route of excretion; BP-3 and its metabolites were excreted mainly as glucuronide conjugates (Kadry et al. 1995; Okereke et al. 1993). Similarly, BP-3 and 2,4-dihydroxylbenzophenone were detected in human urine collected after a volunteer applied a commercially available sunscreen (Felix et al. 1998). These data suggest that the conjugated species of BP-3 and its metabolites in urine can be used as biomarkers of exposure. Oxidative metabolites of BP-3 can themselves be used as sunscreen agents. Although BP-3 can be biotransformed to several metabolites, exposure to BP-3 can be assessed by measuring the total (free plus conjugated) concentrations of BP-3 in urine.

The detection of BP-3 in the aquatic environment and the widespread use of products containing BP-3 have raised interest about assessing human exposure to this compound for risk assessment. We report here the first nationally representative data on the urinary concentrations of BP-3 in the U.S. general population ≥ 6 years of age, stratified by age group, sex, and race/ethnicity.

## Materials and Methods

The National Health and Nutrition Examination Survey (NHANES), conducted continuously since 1999 by the CDC, assesses the health and nutritional status of the civilian noninstitutionalized U.S. population ≥ 2 months of age (CDC 2003). The survey includes household interviews, medical histories, standardized physical examinations, and collection of biologic specimens, some of which can be used to assess exposure to environmental chemicals. NHANES 2003–2004 included examinations of 9,282 people (CDC 2006a). We measured BP-3 by analyzing a random one-third subset of urine samples (*n* = 2,517) collected from NHANES participants ≥ 6 years of age. Because this subset was randomly selected from the entire set, it maintained the representativeness of the survey. Participants provided informed written consent; parents provided informed written consent for their children.

Urine specimens were shipped on dry ice to the CDC’s National Center for Environmental Health and stored frozen at or below –20°C until analyzed. We measured total (free plus conjugated species) concentrations of BP-3 in urine by online solid-phase extraction coupled to high-performance liquid chromatography–tandem mass spectrometry described in detail elsewhere (Ye et al. 2005a). Briefly, conjugated BP-3 in 100 μL of urine was hydrolyzed using β-glucuronidase/ sulfatase (*Helix pomatia*; Sigma Chemical Co., St. Louis, MO). After hydrolysis, samples were acidified with 0.1 M formic acid; BP-3 was preconcentrated by online solid-phase extraction, separated by reversed-phase high-performance liquid chromatography, and detected by atmospheric pressure chemical ionization–tandem mass spectrometry. Because a stable isotope-labeled BP-3 was not available, we used ^13^C_12_-bisphenol A as internal standard (Ye et al. 2005a). The limit of detection (LOD), calculated as 3S_0_, where S_0_ is the standard deviation as the concentration approaches zero (Taylor 1987), was 0.34 μg/L, and the precision ranged from 17.6% (at 18.5 μg/L) to 16.2% (at 46 μg/L). Low-concentration (~ 20 μg/L) and high-concentration (~ 45 μg/L) quality control materials, prepared from pooled human urine, were analyzed with standard, reagent blank, and NHANES samples (Ye et al. 2005a).

We analyzed the data using Statistical Analysis System (version 9.1.3; SAS Institute, Inc., Cary, NC) and SUDAAN (version 9.0.1; Research Triangle Institute, Research Triangle Park, NC). SUDAAN calculates variance estimates after incorporating the sample population weights, nonresponse rates, and sample design effects. We calculated the percentage of detection and the geometric mean and distribution percentiles for both the volume-based (in micrograms per liter urine) and creatinine-corrected (in micrograms per gram creatinine) concentrations. For concentrations below the LOD, as recommended for the analysis of NHANES data (CDC 2006b), we used a value equal to the LOD divided by the square root of 2 (Hornung and Reed 1990).

A composite racial/ethnic variable based on self-reported data defined three major racial/ethnic groups: non-Hispanic black, non-Hispanic white, and Mexican American. We included participants not defined by these racial/ethnic groups only in the total population estimate. Age, reported in years at the last birthday, was stratified in groups (6–11, 12–19, 20–59, and ≥ 60 years of age) for calculation of the geometric mean and the various percentiles.

We used analysis of covariance to examine the influence of several variables, selected on the basis of statistical, demographic, and biologic considerations, on the concentrations of BP-3. For the multiple regression models, we used the variables described below and all possible two-way interactions to calculate the adjusted least square geometric mean (LSGM) concentrations. LSGM concentrations provide geometric mean estimates (in micrograms per liter) after adjustment for all covariates in the model. Because the distributions of BP-3 and creatinine concentrations were skewed, these variables were log transformed. We analyzed two separate models: one for adults (≥ 20 years of age) and one for children and teenagers (≤ 19 years of age). We considered age (continuous), age squared, sex, race/ethnicity, and log-transformed crea-tinine concentration for both models. When the model included both age and age squared, we centered age by subtracting 50 from each participant’s age, thus avoiding multi-collinearity and obtaining the least weighted correlation between these two variables (Bradley and Srivastava 1979). Additionally, to further evaluate the relation between the log-transformed BP-3 concentration and age, we used age group (20–29, 30–39, 40–49, and ≥ 50 years of age) as a categorical variable in the model and generated a bar chart of LSGM concentrations by age group.

To reach the final reduced model, we used backward elimination with a threshold of *p* < 0.05 for retaining the variable in the model, using Satterwaite-adjusted *F* statistics. We evaluated for potential confounding by adding each of the excluded variables back into the final model one by one and examining changes in the β coefficients of the statistically significant main effects or interactions. If addition of one of these excluded variables caused a change in a β coefficient by ≥ 10%, we re-added the variable to the model.

We also conducted weighted univariate and multiple logistic regressions to examine the association of BP-3 concentrations above the 95th percentile with sex, age group, and race/ ethnicity for all ages.

## Results

We detected BP-3 in 96.8% of the 2,517 samples at concentrations ranging from 0.4 to 21,700 μg/L; the geometric mean and 95th percentile concentrations were 22.9 μg/L (22.2 μg/g creatinine) and 1,040 μg/L (1,070 μg/g creatinine), respectively (Table 1).

**Figure 1:**
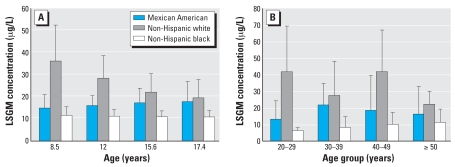
LSGM concentrations of BP-3 (in micrograms per liter) by age and race/ethnicity: (*A*) children and adolescents and (*B*) adults. Error bars indicate 95% CIs.

The final model for adults included sex, race/ethnicity, age, age squared (*p* = 0.038), creatinine concentration (log scale), and the interaction terms creatinine*sex (*p* < 0.001) and age*race/ethnicity (*p* = 0.04) (Table 2). Females had significantly higher BP-3 concentrations (*p* ≤ 0.04) than did males, regardless of creatinine level [see Supplemental Tables S1 and S2 (http://www.ehponline.org/members/2008/11269/suppl.pdf). Although BP-3 concentrations increased linearly as log creatinine increased for both sexes (*p* < 0.001), the increase was more pronounced for males than for females (β for males, 1.12; for females, 0.65). Also, as age increased, BP-3 LSGM concentrations showed a significant quadratic trend for Mexican Americans (*p* = 0.016) and a significant linear positive trend for non-Hispanic blacks (*p* = 0.022) but no significant linear or quadratic trend for non-Hispanic whites (Figure 1). LSGM concentrations of BP-3 for non-Hispanic whites were significantly higher than for non-Hispanic blacks, regardless of age (*p* ≤ 0.01), and significantly higher than for Mexican Americans only for 20- to 29-year-olds (*p* = 0.01). LSGM concentrations of BP-3 were significantly higher for Mexican Americans than for non-Hispanic blacks only for 30- to 39-year-olds (*p* = 0.01) [see Supplemental Tables S1 and S2 (http://www.ehponline.org/members/2008/11269/suppl.pdf)].

The final model for children and adolescents included sex (*p* < 0.001), race/ethnicity, age, creatinine concentration (log scale) (*p* < 0.001), and a race/ethnicity*age (*p* = 0.01) interaction term (Table 2). LSGM concentrations of BP-3 increased as log creatinine increased (β = 0.77, *p* < 0.001). LSGM BP-3 concentrations for girls [30.2 μg/L; 95% confidence interval (CI), 21.4–42.6 μg/L] were significantly higher (*p* < 0.001) than for boys (16.1 μg/L; 95% CI, 13.2–19.8 μg/L). BP-3 concentrations also decreased linearly as age increased (*p* = 0.0005) for non-Hispanic whites but not for Mexican Americans and non-Hispanic blacks [Figure 1; see also Supplemental Table S3 (http://www.ehponline.org/members/2008/11269/suppl.pdf)]. LSGM concentrations of BP-3 for non-Hispanic whites were significantly higher than LSGM concentrations for non-Hispanic blacks, regardless of age, and for Mexican Americans only at younger ages [*p* < 0.001 at 8.5 years, *p* < 0.01 at 12 years; Supplemental Table S4 (http://www.ehponline.org/members/2008/11269/suppl.pdf)]. LSGM BP-3 concentrations were significantly higher for Mexican Americans than for non-Hispanic blacks only for older children (*p* = 0.01, at 12 and at 15.6 years, *p* = 0.03 at 17.4 years) [Supplemental Table S4 (http://www.ehponline.org/members/2008/11269/suppl.pdf)].

For participants with urinary concentrations above the 95th percentile of BP-3, sex (*p* < 0.001) and race/ethnicity (*p* = 0.03), but not age, were significantly associated univariately. In the final multiple logistics regression, sex (*p* < 0.001) and race/ethnicity (*p* = 0.03) were significant [Supplemental Table S5 (http://www.ehponline.org/members/2008/11269/suppl.pdf)]. Females were 3.5 times more likely than males to be above the 95th percentile [adjusted odds ratio (OR) = 3.5; 95% CI, 1.9–6.5]. Non-Hispanic whites were 6.8 times more likely to have BP-3 concentrations above the 95th percentile (adjusted OR = 6.8; 95% CI, 2.9–16.2) than were non-Hispanic blacks, and Mexican Americans were four times more likely to be above the 95th percentile (adjusted OR = 4.04; 95% CI, 1.1–15.5) than were non-Hispanic blacks. We found no significant difference between non-Hispanic whites and Mexican Americans.

## Discussion

The detection of BP-3 in almost all samples suggests that exposure to BP-3 was widespread in the U.S. general population during 2003–2004. This high level of detection most likely resulted from routine use of consumer products that contain BP-3, such as sunscreen, skin care lotion, lipstick, and hair spray (National Library of Medicine 2007). The wide range of urinary concentrations—10% of participants had BP-3 concentrations < 2.3 μg/g creatinine and 5% had concentrations > 1,070 μg/g creatinine (Table 1)—may be related to lifestyle differences that result in exposure differences and to individual variations in bioavailability, distribution kinetics, or metabolism of BP-3.

The frequent detection of BP-3 and the magnitude and range of urinary concentrations in NHANES 2003–2004 are comparable with data from two smaller studies in the United States. In 30 anonymous adult volunteers with no documented BP-3 exposure, we detected BP-3 in 90% of samples, and total urinary concentration (free plus conjugates) of BP-3 ranged from the LOD (0.5 μg/L) to 3,000 μg/L (Ye et al. 2005b). In a pilot study of 90 prepubertal girls from New York City, New York; Cincinnati, Ohio; and Northern California, we detected BP-3 in 86% of samples (Wolff et al. 2007). The creatinine-adjusted geometric mean concentration of BP-3 (30.8 μg/g) for these girls was similar to that for NHANES 2003–2004 children 6–11 years of age (25.8 μg/g creatinine).

The relation between age and LSGM BP-3 concentrations differed by race/ethnicity (Figure 1). These differences most likely result from increased use of sunscreen or other personal-care products containing BP-3 by people with light skin pigmentation. For instance, sunscreen use among non-Hispanic whites is reportedly higher than for non-Hispanic blacks and other race/ethnic groups of outdoor workers and the general population (Briley et al. 2007; Pichon et al. 2005). Likewise, differences by age might reflect differences in use of personal-care products that contain BP-3. Non-Hispanic white parents may apply sun-screen regularly to protect their young children from sunburn, whereas teenagers might not apply sunscreen as often (Jones and Saraiya 2006; Livingston et al. 2007). Non-Hispanic white adults in their twenties and forties might be more preoccupied about their skin appearance than non-Hispanic whites in their thirties (who may devote more time to work and family responsibilities than to themselves) or people in their fifties (who may see little benefit in applying sunscreen at older ages).

We found differences by sex in the adjusted LSGM concentrations of BP-3. Compared with males, females tend to use more sunscreen (Eide and Weinstock 2006; Hall et al. 1997; Jones and Saraiya 2006) and other personal-care products that may contain BP-3. Therefore, higher concentrations of BP-3 for females than for males most likely result from their higher exposure to BP-3.

Females and non-Hispanic whites not only had significantly higher LSGM concentrations than did males and non-Hispanic blacks, respectively, but also were more likely to exhibit concentrations of BP-3 above the 95th percentile. In particular, females were 3.5 times more likely than males, and non-Hispanic whites were 6.8 times more likely than non-Hispanic blacks to have BP-3 concentrations above the 95th percentile. Mexican Americans were about four times more likely than non-Hispanic blacks to present BP-3 concentrations above the 95th percentile. Although young children had LSGM concentrations of BP-3 comparable with those of adults in their twenties and forties, age was not significantly associated with having concentrations above the 95th percentile. Our data suggest that females and non-Hispanic whites represent two segments of the general population with higher exposures to BP-3 compared with other demographic groups.

Protection against sunburn and squamous cell carcinoma by application of sunscreens is important, even though the use of sunscreen may not protect against melanoma, the deadliest form of skin cancer (Lin and Fisher 2007). Sun protection is critical for outdoor workers, who are at higher risk for squamous cell carcinoma than other population groups (Pichon et al. 2005), and in situations where sun exposure, even during peak times, is unavoidable. In other situations, although behavioral measures, such as wearing a hat, sunglasses, and sun protective clothes and avoiding the sun during peak exposure times, can reduce the risk for skin damage, sunscreens may be the primary means of sun protection, especially in societies that value outdoor activities (Lautenschlager et al. 2007). Toxicologic and epidemiologic data on BP-3, one of these sunscreens, are lacking. Nevertheless, the NHANES 2003–2004 data demonstrating Americans’ exposure to BP-3 can be used to establish a nationally representative baseline assessment of exposure to this sunscreen agent and may promote the use of biomonitoring to complement the questionnaire or survey information in studies designed to evaluate sun-safety practices. These NHANES 2003–2004 data could also be of benefit in a risk assessment for BP-3 if indicated by toxicologic or epidemiologic studies.

## Figures and Tables

**Table 1:** Geometric mean and selected percentiles of BP-3 concentrations in urine for the U.S. population ≥ 6 years of age: data from NHANES 2003–2004.^a^

		Selected percentile						
Variable^b^	Geometric mean	10th	25th	50th	75th	90th	95th	Sample (*n*)
Total	22.9 (18.1–28.9)	2.20 (1.50–2.60)	5.80 (4.70–7.10)	18.0 (15.3–23.1)	94.0 (67.5–123)	364 (225–570)	1,040 (698–1,390)	2,517
	22.2 (17.6–28.0)	2.28 (1.73–2.89)	5.24 (4.27–6.21)	16.2 (12.7–21.6)	82.0 (58.7–108)	409 (283–577)	1,070 (686–1,600)	2,514
Age group (years)								
6–11	21.2 (16.4–27.3)	3.60 (2.40–4.50)	6.70 (5.20–9.50)	17.2 (14.9–25.9)	63.6 (38.7–102)	154 (106–246)	227 (154–618)	314
	25.8 (19.5–34.1)	4.30 (2.86–5.19)	8.25 (5.98–10.5)	22.4 (14.4–33.7)	83.6 (41.0–131)	171 (132–365)	427 (171–710)	314
12–19	22.9 (18.0–29.3)	3.30 (2.30–4.10)	7.80 (5.60–9.60)	20.0 (16.1–25.1)	66.5 (45.2–93.8)	170 (137–240)	407 (183–717)	715
	17.2 (13.7–21.5)	3.17 (2.24–4.03)	5.86 (4.81–6.93)	12.9 (10.3–16.5)	42.9 (29.5–57.7)	136 (91.7–239)	350 (173–646)	713
≥ 20	23.1 (18.0–29.6)	1.80 (1.20–2.40)	5.50 (4.50–6.70)	18.1 (14.7–23.3)	108 (72.1–140)	450 (315–733)	1,200 (769–1,750)	1,488
	22.8 (17.8–29.1)	1.98 (1.48–2.59)	4.89 (3.71–6.12)	16.2 (12.7–21.9)	93.2 (66.0–130)	486 (361–700)	1,330 (880–1,880)	1,487
Sex								
Female	30.7 (23.7–39.8)	2.50 (1.80–3.40)	7.30 (5.40–9.10)	26.0 (20.2–34.1)	137 (105–172)	596 (403–769)	1,340 (776–1,790)	1,288
	35.5 (27.1–46.4)	3.16 (2.28–4.13)	7.42 (5.83–9.39)	28.2 (20.2–37.0)	144 (101–224)	686 (491–1,130)	1,850 (1,220–2,580)	1,286
Male	16.8 (13.2–21.3)	1.80 (1.30–2.20)	5.00 (4.30–5.90)	13.6 (11.4–16.8)	54.4 (33.2–86.5)	178 (134–324)	567 (238–1,350)	1,229
	13.6 (10.8–17.1)	1.82 (1.55–2.16)	3.81 (3.33–4.87)	10.2 (8.36–12.9)	40.0 (24.9–62.5)	169 (93.3–316)	378 (229–685)	1,228
Race/ethnicity								
Non-Hispanic white	27.7 (20.3–37.8)	2.30 (1.50–3.00)	6.80 (5.10–8.60)	23.5 (16.8–32.0)	120 (83.6–162)	501 (316–769)	1,250 (733–2,070)	1,092
	28.3 (20.6–38.8)	2.55 (1.80–3.62)	6.07 (4.88–8.33)	21.9 (14.6–32.7)	116 (73.5–175)	510 (380–760)	1,330 (852–2,410)	1,091
Mexican American	16.5 (10.9–25.1)	2.30 (1.70–3.70)	5.00 (3.70–6.60)	11.9 (8.30–18.3)	45.5 (25.9–78.2)	176 (68.7–346)	412 (178–2,180)	613
	15.1 (9.44–24.0)	2.39 (1.68–3.26)	4.10 (2.95–6.71)	11.0 (6.95–16.0)	40.7 (18.3–85.8)	158 (87.4–362)	595 (118–1,860)	612
Non-Hispanic black	12.8 (9.38–17.4)	2.10 (1.30–2.70)	4.60 (3.20–6.20)	10.2 (7.40–14.3)	34.2 (22.8–50.6)	127 (90.8–176)	209 (143–499)	652
	8.78 (6.49–11.9)	1.50 (1.05–2.35)	3.18 (2.42–4.14)	6.80 (5.27–9.00)	19.6 (13.5–33.4)	78.1 (46.8–139)	185 (79.8–536)	651

a Concentrations are given as micrograms per liter (unshaded) and micrograms per gram creatinine (shaded), with 95% CIs in parentheses.

b Participants not defined by the three racial/ethnic groups shown were included only in the total population estimate.

**Table 2:** Coefficients for the significant variables from the multiple regression models of the BP-3 urinary concentration (log-transformed) by age group [β coefficient (*p*-value)].

Variable	Children and adolescents (6–19 years of age)	Adults (≥ 20 years of age)
Intercept	−0.33985 (0.14913)	−0.08999 (0.73675)
Sex		
Male	−0.27143 (0.00079)	−1.39213 (0.00079)
Female	Reference	
Race/ethnicity		
Mexican American	0.01857 (0.91283)	0.24104 (0.15686)
Non-Hispanic white	0.73888 (0.00035)	0.48352 (0.00001)
Non-Hispanic black	Reference	
Age	−0.00243 (0.73485)	0.00155 (0.50696)^a^
Creatinine concentration (log transformed)	0.76653 (<0.001)	0.64519 (0.00008)
Age squared (centered)		−0.00018 (0.03848)
Race/ethnicity*age		
Mexican American	0.01139 (0.4116)	−0.00365 (0.2125)^a^
Non-Hispanic white	−0.02787 (0.0249)	−0.00784 (0.02026)^a^
Non-Hispanic black	Reference	
Sex*log creatinine		
Male		0.47423 (0.00811)
Female		Reference

a Age centered at 50 years.
